# Assessing single camera markerless motion capture with OpenSim inverse kinematics during upper limb activities of daily living

**DOI:** 10.1080/23335432.2025.2556187

**Published:** 2025-09-05

**Authors:** Bradley Scott, Mhairi McInnes, Edward K. Chadwick, Dimitra Blana

**Affiliations:** a School of Medicine, Medical Sciences and Nutrition, University of Aberdeen, Aberdeen, UK; b School of Engineering, University of Aberdeen, Aberdeen, UK

**Keywords:** Markerless, Azure Kinect, inverse kinematics, OpenSim, upper limb analysis

## Abstract

This study evaluates the accuracy of single camera markerless motion capture (SCMoCap) using Microsoft’s Azure Kinect, enhanced with inverse kinematics (IK) via OpenSim, for upper limb movement analysis. Twelve healthy adults performed ten upper-limb tasks, recorded simultaneously by OptiTrack (marker-based) and Azure Kinect (markerless) from frontal and sagittal views. Joint angles were calculated using two methods: (1) direct kinematics based on body coordinate frames and (2) inverse kinematics using OpenSim’s IK tool with anatomical keypoints. Accuracy was evaluated using root mean square error (RMSE) and Bland-Altman analysis. Results indicated that the IK method slightly improved joint angle agreement with OptiTrack for simpler movements, with an average RMSE of 8° for shoulder elevation in the sagittal plane compared to 9° with the coordinate frame method. However, both methods had higher RMSEs for rotational measurements, with IK and coordinate frame methods at 21° for shoulder rotation in the sagittal plane. Forearm pronation-supination measurements were unreliable due to tracking limitations. These findings suggest that Kinect with IK improves accuracy for simpler movements but struggles with rotational joint mechanics. Future research should focus on enhancing markerless tracking algorithms to fully realise the benefits of IK.

## 1. Introduction

Three-dimensional motion capture has been foundational in biomechanical analysis for several decades (Topka et al. 1998; Corazza et al. 2006; Noort et al. 2016). This technology enables precise quantification and identification of complex human movement patterns and impairments. For example, when integrated into clinical interventions, such as hamstring lengthening surgery for children with cerebral palsy, it provides crucial biomechanical insights that enhance patient outcomes and inform clinical decision-making (Arnold et al. 2006; Laracca et al. 2014). Currently, the predominant approach in clinical settings is marker-based motion capture, in which reflective markers are placed on the patient’s body and then tracked by multiple cameras. While this clinical motion capture offers several benefits to patients and clinicians, including reducing unnecessary surgeries and healthcare costs (Salami et al. 2019; Osborne et al. 2019), the practical and logistical demands of the marker-based approach often limit its use in clinical practice (Philp et al. 2021). Clinical motion capture laboratories require substantial infrastructure, including multiple cameras within large capture volumes, well-defined marker sets, and skilled technical staff. These requirements centralise the technology in specialised centres, limiting access for many patients. Furthermore, these specialised centres primarily focus on analysing gait, as capturing upper limb movement presents challenges, such as marker occlusion and a lack of standardized reference tasks (Philp et al. 2022).

In recent years, there has been growing interest in *markerless* motion capture systems, which eliminate the need for marker attachment and offer a less intrusive assessment for patients. However, markerless systems which have comparable accuracy to marker-based setups, such as Theia3D (Theia Markerless Inc., Kingston, ON, Canada; Kanko et al. (2021) and OpenCap (Uhlrich et al. 2023), still have limitations: they generally require multiple cameras, complex calibration for home settings, and come at a high cost. *Single* camera markerless motion capture (SCMoCap) systems, such as Microsoft’s Azure Kinect (Microsoft, USA), have emerged as a more promising alternative to marker-based methods. With their quicker set-up time, portability, and affordability, these systems could enable motion analysis in more clinical settings and reduce the need for travel to specialised centers, which can be challenging for patients with limited mobility. The Azure Kinect was selected for this study because it offers measured depth data, unlike other single-camera 2D-to-3D lifting methods that rely on estimated depth, and serves as an example depth sensing system, with the proposed methodology extensible to other depth-based devices. In a recent scoping review (Scott et al. 2022), discussed the potential for SCMoCap in a range of healthcare scenarios, from supporting at-home rehabilitation for children with cerebral palsy (Kidziński Yang et al. 2020) to monitoring falls in the homes of older adults (Stone and Skubic 2015).

Despite the potential of SCMoCap, the review highlighted several gaps in the literature relating to accuracy and clinical interpretability. Studies to date have focused mainly on population discrimination (Sá et al. 2015; Eltoukhy et al. 2017) or range of motion calculations (Galna et al. 2014; Capecci et al. 2018; Özsoy et al. 2022; Van Crombrugge et al. 2022), which offer only partial insights into the accuracy of joint angle measurements. Many studies report repeatability with statistical measures that lack clinical interpretability, and few evaluate the full workspace of the shoulder and elbow, instead focusing on gait. In addition, there was a lack of synchronous data collection for the Kinect, which likely led to suboptimal comparisons between reference and markerless methods due to the inherent intra-subject variability in upper limb movement patterns. Furthermore, no studies were found that utilised the Kinect’s joint coordinate systems, relying instead on joint keypoints alone. Finally, no studies have employed inverse kinematics and a musculoskeletal model to calculate joint angles, focusing only on direct-kinematics which may limit anatomical precision and prevent the correct handling of joint mechanics.

The motivation for this work arises from these limitations in the current literature. Providing a comprehensive validation of SCMoCap, alongside clinically meaningful evaluations, would benefit clinicians and researchers interested in implementing upper limb movement analysis in clinical practice. To address these gaps, we explore the potential of inverse kinematics (IK) through OpenSim (Delp et al. 2007), an open-source biomechanical modeling software, to improve estimates of joint kinematics. Using a musculoskeletal model, OpenSim’s IK tool aims to calculate the pose of the model by minimising the error between predicted markers and virtual model markers. The error can be distributed across markers, and the mechanical restrictions of the model’s joint can be strictly enforced. This approach may be especially beneficial in single camera setups, where occlusion and 2D-to-3D depth estimation challenges can affect accuracy (Zheng et al. 2023). Additionally, camera positioning may influence the accuracy of joint angle measurements due to variations in occlusion and depth estimation across different perspectives (Seo et al. 2016). In light of these considerations, our work addresses the following questions: To what extent does the Azure Kinect agree with marker-based motion capture systems in the measurement of joint angles during upper limb activities of daily living? Can inverse kinematics improve the joint angle accuracy of single-camera markerless motion capture? And finally, what position of the Azure Kinect provides the best agreement?

## 2. Methods

To evaluate the accuracy of the Azure Kinect, we conducted an experimental trial with *12* participants. Each participant performed *10* upper-limb movements while being recorded simultaneously by an optoelectronic motion capture system (OptiTrack) and the Azure Kinect in the University of Aberdeen biomechanics laboratory. These movements were captured from both a frontal and a sagittal viewpoint, with each movement repeated for both views. The following sections detail participant demographics, the marker-based and markerless setups, synchronisation methods, the movements performed, joint angle calculations, and the statistical analyses used to compare the two systems.

### 2.1. Participant demographics

Eligible participants for the study were healthy adults with no recent movement-restricting injuries or history of neurological or musculoskeletal disorders, and no age restrictions. Ethical approval was granted by the University of Aberdeen Physical Sciences and Engineering Ethics Board, and all data collection was carried out with informed consent.

### 2.2. Marker-based setup

**Figure 1.**
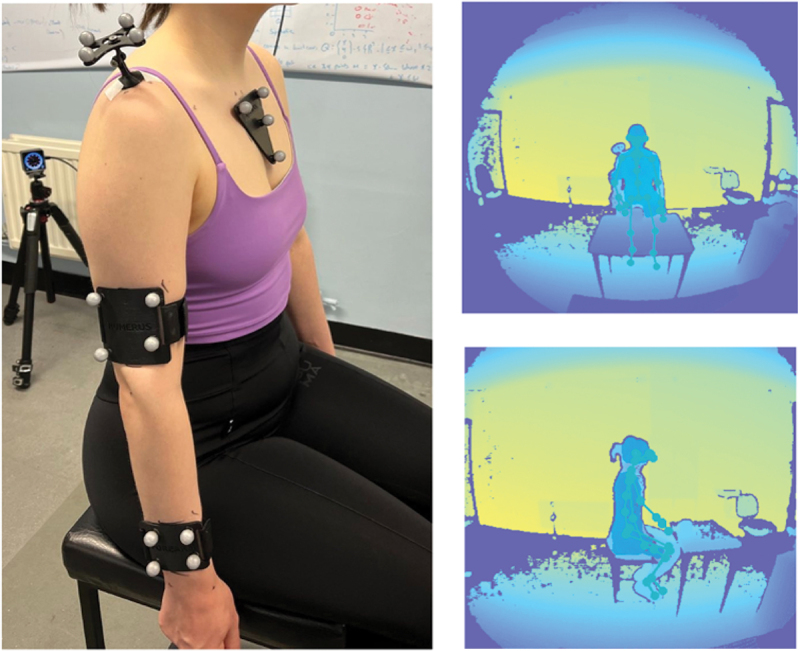
Example participant setup for marker-based and markerless.

A 10-camera OptiTrack prime 13W passive retroreflective system was used for the marker-based motion capture. To ensure tracking accuracy and to mitigate issues with self-occlusion, 3D-printed marker-clusters were positioned on the sternum, acromion, upper arm, and forearm, with 10 mm markers attached, in a pattern which was unique and non-collinear (see Figure 1). Sternum and acromion marker clusters were attached using double-sided medical tape; upper arm and forearm markers were attached distally (to optimally capture rotation) using adjustable hook-and-loop fasteners (to minimise the movement of the cluster and for the participant’s comfort). The edges of each cluster were highlighted with a makeup pencil and this boundary was checked periodically throughout the trial to mitigate any movements of the clusters. It was essential to place the markers a few centimetres away from the joint centers to prevent any potential interference with the Kinect body tracking software. The OptiTrack system was configured within the Motive software (2.3.0). Exposure settings for each camera were adjusted through an iterative process, involving visual inspection of markers to ensure optimal visibility within the capture area. The capture volume, free from markers, was masked to handle any unwanted reflections. The OptiTrack system was calibrated using a calibration wand, achieving an ‘exceptional’ reprojection error level with a mean 3D error below 1 mm. Finally, the world axis and ground plane were established using a calibration square. Marker-based data were recorded using Motion Monitor v.3.55.7.0 (MM) at 120 Hz.

Each subject was calibrated by recording the position of bony landmarks with a marker-mounted pointer. With the subject in the neutral pose, the following landmarks were palpated and their positions marked on the skin with a makeup pencil: Thorax (C7 (seventh cervical vertebra), T8 (eighth thoracic vertebra), Incisura Jugularis (IJ), Processus Xiphoideus (PX), Clavicle (Sternoclavicular Joint (SC), Acromioclavicular Joint (AC)), Scapula (Trigonum Spinae Scapulae (TS), Angulus Inferior (AI), Angulus Acromialis (AA), Processus Coracoideus (PC)), Humerus (Glenohumeral rotation center (GH), lateral epicondyle (EL), medial epicondyle (EM)), and Forearm (radial styloid (RS), ulnar styloid (US). Remaining in the neutral position, the position of each landmark was recorded with a pointer, providing a permanent definition of the skeletal coordinate frames, relative to the marker clusters. The glenohumeral joint center was estimated from rotational movement, using the centre transformation technique (Ehrig et al. 2006). Bone segment coordinate frame were defined with the Motion Monitor software, following the definitions outlined by the International Society of Biomechanics (Wu et al. 2005).

### 2.3. Single camera markerless setup

For the single-camera markerless setup, an Azure Kinect was placed on a tripod at 1.5 m either in front of the participant or to the side, providing either a frontal and sagittal view during movements (see Figure 1). The Azure Kinect was chosen as it provides measured depth through its time-of-flight IR camera, as opposed to 2D to 3D lifting typically used in many single-camera algorithms (Ben Gamra and Akhloufi 2021). The Azure Kinect body tracking software was used to perform markerless pose estimation and extract joint coordinate frames and keypoints at 30 Hz captured with W-FOV depth mode and RGB 1080p. W-FOV was required to ensure all movements were in-frame. To ensure that the entire movement was captured the Kinect height was adjusted via the tripod for each participant as they extended their arms around the capture volume. To assess the impact of camera placement on measurement accuracy, movements were recorded separately from frontal and sagittal views, selected for their convenience in clinical and home settings due to straightforward setup and minimal space requirements.

### 2.4. Time synchronisation of maker-based and markerless data collection

The Kinect’s infrared sensor was affected by IR multipath interference (Naeemabadi et al. 2018) from the OptiTrack cameras. To ensure synchronous data capture, an eSync2 device (OptiTrack, USA) was used to pulse OptiTrack’s IR signals out of phase with the Kinect’s, preventing interference. To achieve this, the Kinect was setup as a master device, and the OptiTrack was a subordinate.

### 2.5. Movements

Upper limb motion was recorded during 10 distinct movements, selected to span the complete workspace of the shoulder and elbow. These movements were divided into two categories: five functional tasks, representing typical activities of daily living often used as clinical outcome measures, such as those in the Barthel Index (Wade and Collin 1988), and five physiological movements designed to isolate specific joint angles. The functional tasks included ‘Brush Hair’, ‘Perineal Care’, ‘Collect Change’, ‘Drink From Cup’, and ‘Eat With Spoon’. The physiological movements comprised ‘Shoulder Abduction’, ‘Shoulder Flexion’, ‘Elbow Flexion’, ‘Shoulder Rotation’, and ‘Pronation Supination’. Each movement was demonstrated to the participant prior to each trial and performed unilaterally with the right arm, repeated three times from both frontal and sagittal viewpoints. Participants were instructed to perform movements at a slow pace, ‘as if they were holding a small weight’, to ensure temporal consistency. All movements were recorded with participants seated for their comfort, and functional tasks utilized props to encourage natural movement execution.

For the functional tasks, a table was placed within the capture workspace in front of the seated participant, with relevant objects (e.g. hairbrush, spoon, cup, or change) positioned on the table and removed after each task to maintain a clear workspace. All functional tasks began with participants positioning their arms down by their sides without flexing the elbow. In the ‘Brush Hair’ task, participants reached forward to grasp a hairbrush from the table, pretended to brush their hair by covering the entire scalp, and then returned the brush to the table. For the ‘Perineal Care’ task, participants reached their arm behind their torso to simulate wiping and then returned to the starting position. In the ‘Collect Change’ task, participants swept change into their hand from the table, placed the change in their pocket, and returned to the starting position. For the ‘Drink From Cup’ task, participants reached forward to grasp an empty cup from the table, brought it to their mouth as if drinking, and returned the cup to the table. In the ‘Eat With Spoon’ task, participants reached forward to grasp an empty spoon from the table, pretended to eat with it, and returned the spoon to the table. These tasks were performed naturally without strict pose instructions to reflect typical activities of daily living, as assessed in the Barthel Index for post-stroke functional evaluation.

The physiological movements were performed with precise instructions to ensure no compensatory movements contributed to the action, aligning with assessments such as the Fugl-Meyer stroke assessment. In the ‘Shoulder Abduction’ task, participants started with their arms down by their sides without flexing the elbow. They were instructed to raise their arm laterally as far as possible without bending the elbow and then lower it back to the starting position. For the ‘Shoulder Flexion’ task, participants began with their arms down by their sides without flexing the elbow. They raised their arm forward in front of their torso as high as possible without bending the elbow, returned to the starting position, then raised their arm backward behind their torso as high as possible without bending the elbow, and returned to the starting position. In the ‘Elbow Flexion’ task, participants started with their arms down by their sides without flexing the elbow, bent their elbow toward the torso, and returned to the starting position. For the ‘Shoulder Rotation’ task, participants began with their arms down by their sides, elbow flexed at 90 degrees with the thumb facing upward. They rotated their upper arm toward their torso as far as possible, returned to the starting position, then rotated their upper arm away from their torso as far as possible, and returned to the starting position. In the ‘Pronation Supination’ task, participants started with their arms down by their sides, elbow flexed at 90 degrees with the thumb facing upward. They rotated their forearm toward the ground as far as possible, returned to the starting position, then rotated their forearm toward the ceiling as far as possible, and returned to the starting position. These controlled movements ensured precise isolation of joint angles for accurate assessment.

### 2.6. Joint angle calculations

Two methods were used to calculate physiological joint angles, specifically shoulder angle of elevation, shoulder plane of elevation, shoulder rotation, elbow flexion-extension, forearm pronation-supination, for comparison in the analysis. The Coordinate (CF) method was applied only to the Kinect data, while the Inverse Kinematics (IK) method was applied to both the Kinect and OptiTrack data. In the first method (CF), quaternions representing segment orientations were extracted from the Azure Kinect body tracking software, then smoothed using Spherical Linear Interpolation (SLERP). Kinect quaternions were converted to rotation matrices, rotated to match the ISB joint coordinate system definitions, and upsampled from 30 Hz to 120 Hz to match the OptiTrack sampling rate. Joint rotation matrices were calculated to represent thorax-to-humerus and humerus-to-forearm rotations, then converted to intrinsic Euler angles, with signs unwrapped to maintain angle continuity.

In the second method (IK), keypoints were used instead of joint coordinate frames. Keypoints were extracted from the Azure Kinect body tracking software. These keypoints, which represented the 3D position of each joint centre, were used as the input to an inverse kinematics solver within OpenSim 4.4. An upper limb model, called the ‘Dynamic Arm Simulator’, or DAS3 model (Chadwick et al. 2014), was used which has a thorax, clavicle, scapula, humerus, radius, and ulna. The thorax, clavicle, scapula and humerus are connected by a series of 3-degree-of-freedom joints, and elbow flexion and forearm pronation is modelled as 1-degree-of-freedom joints. Each joint was constrained to a realistic range of motion. New virtual markers were defined within the model to match the keypoints of the Kinect data (e.g. wrist joint keypoint was the centre-point of US and RS). For each participant, the model was scaled based on the distances between recorded keypoints, then the inverse kinematics tool was used to calculate the pose of the model which minimised the Euclidean distance between the virtual and estimated keypoints. The joint angles reported from the model were clinically interpretable and based on ISB standards. Marker coordinates were smoothed using a fourth-order, zero-phase Butterworth filter (5 Hz cutoff, 30 Hz sampling). The Kinect data were then upsampled to 120 Hz to match the marker-based sampling rate. For both methods, cross-correlation was applied to the joint angle data to time-synchronise the Kinect and marker-based joint angles. This was necessary because the eSync2 device only synchronised the IR signal pulses between the Kinect and OptiTrack to prevent multipath interference, not the data signals themselves.

Reference joint angles for the OptiTrack (marker-based) data were calculated using OpenSim inverse kinematics, following a procedure similar to the IK method but without upsampling or creating new model markers, instead utilising the provided DAS3 markers. Marker coordinates were smoothed using a fourth-order, zero-phase Butterworth filter with an 8 Hz cutoff and a 120 Hz sampling rate.

### 2.7. Statistical analysis

To evaluate the agreement of the Azure Kinect compared to marker-based motion capture (OptiTrack) for measuring joint angles during upper limb activities of daily living (ADLs), statistical analyses were conducted to address the research questions, using a repeated measures Bland-Altman analysis to quantify agreement and one-way ANOVA to assess statistical significance of differences in Root Mean Square Error (RMSE). Results were reported by joint angle and averaged by plane, angle type, and task type (all tasks, planar movements, or ADLs) across 12 participants to facilitate clinical interpretation and systematically compare coordinate frame and inverse kinematics methods against OptiTrack, as well as sagittal and frontal viewpoints for each category.

Forearm pronation-supination was excluded from the analysis because the CF method could not accurately measure it. This was due to the elbow’s lateral axis aligning with the shoulder’s, as verified by a dot product test confirming only five independent axes. However, the shoulder and chest axes still allowed estimation of shoulder rotation. Similarly, the IK method failed to accurately calculate pronation-supination because of poor thumb marker tracking. As a global optimization method, this affected other joint angles. Consequently, pronation-supination was locked in the model and the thumb marker was excluded (see Supplementary Figure S1: Example angle agreement for collect change movement recorded viewing the sagittal plane (IK)). As a result, only four angle sets were analysed: plane of elevation, angle of elevation, shoulder rotation, and elbow flexion-extension.

#### 2.7.1. Repeated measures Bland-Altman analysis and RMSE for agreement

To address the first research question, which evaluates the extent of agreement between Azure Kinect and marker-based motion capture (OptiTrack), a repeated measures Bland-Altman analysis, following the methodology of Wade et al. (2023), was used to quantify systematic bias and limits of agreement (LoA) between Kinect-based methods (CF or IK) and OptiTrack measurements. The goal of this analysis was to assess agreement by calculating the mean difference (bias) and the 95% confidence interval (LoA) for the differences between methods. Agreement was evaluated for each combination of plane (sagittal or frontal), movement (10 movements, e.g. drink from cup, shoulder abduction), and angle set (4 angle sets: plane of elevation, angle of elevation, shoulder rotation, elbow flexion-extension), corresponding to each angle difference set. Independent Bland-Altman analyses were conducted for each method (coordinate frame and inverse kinematics) within each plane/movement/angle set combination, with each method having 80 difference sets (2 planes ×10 movements ×4 angle sets).

For each difference set, a one-way analysis of variance (ANOVA) was applied with Participant (12 participants per difference set) as the independent variable (factor) and Angle Difference (the difference between coordinate frame or inverse kinematics and marker-based measurements) as the dependent variable to partition the variance into between-participant and within-participant (residual) components. The between-participant variance was calculated as the difference between the mean square participant and mean square residual, divided by the average number of observations per participant, and the total variance was the sum of this between-participant variance and the within-participant variance (mean square residual). The standard deviation (SD) was computed as the square root of this total variance.

For the Bland-Altman analysis, the bias was derived as the mean of the angle differences across all 12 participants for each difference set, and the LoA were calculated as the bias ±1.96 ×SD, representing the 95% confidence interval for agreement between each method (CF or IK) and the reference (OptiTrack). The RMSE, providing a measure of within-participant variability, was derived as the square root of the mean square residual from this ANOVA for each plane/movement/angle set combination.

#### 2.7.2. ANOVA tests for method and Kinect position comparisons

To address the second and third research questions, which investigate whether inverse kinematics improves joint angle accuracy and which Azure Kinect position (sagittal or frontal plane) provides the best agreement, four one-way ANOVA tests were conducted with a significance level of α=0.05, including two for method comparisons (one per plane) and two for plane comparisons (one per method). These ANOVA tests used RMSE, derived from the variance partitioning in the Bland-Altman analysis, to assess whether differences in measurement accuracy between methods and between planes were statistically significant.

For the second research question, a one-way ANOVA was performed for each plane (sagittal and frontal) to determine whether the mean RMSE differed significantly between IK and CF, with Method as the independent variable (factor) and RMSE as the dependent variable, using values pooled across the 40 difference sets (10 movements ×4 angle sets) per plane per method.

For the third research question, a one-way ANOVA was performed for each method to determine whether the mean RMSE differed significantly between the sagittal and frontal planes, with Plane as the independent variable (factor) and RMSE as the dependent variable, comparing the 40 sagittal difference sets (10 movements ×4 angle sets) against the 40 frontal difference sets (10 movements ×4 angle sets) for that method. For each ANOVA, the F-statistic, p-value, and Cohen’s f, derived from partial eta-squared, were calculated to assess statistical significance and effect size.

## 3. Results

This study included 12 participants (6 males, 6 females) aged 22–33 years (mean = 28, SD = 4), with heights ranging from 156 to 193 cm (mean = 173, SD = 11) and weights ranging from 58 to 100 kg (mean = 73, SD = 14).

Table 1 shows the results for joint angle agreement for both CF and IK methods averaged for each angle and task type, and recorded viewpoint. For a full list of movements and their corresponding agreements, see Supplementary Table S1 for the coordinate frame method and Supplementary Table S2 for the inverse kinematics method.

**Table 1.** Average joint angle agreement between Azure Kinect and OptiTrack for frontal and sagittal planes, comparing coordinate frame and OpenSim IK methods.

Plane	Angle Type	Task Type	Bias (°) [SD]		Lower LOA (°)		Upper LOA (°)		RMSE (°)	
			CF	IK	CF	IK	CF	IK	CF	IK
Frontal Average		All Tasks	0.4 (21.3)	2.1 (23)	−41.4	−43	42.4	47.1	17.4	16.7
Sagittal Average		All Tasks	0.4 (19.8)	5.3 (22)	−38.3	−37.9	39	48.6	15.6	14.8
Frontal	Angle Of Elevation	All Tasks	6.5	3.5	−21.4	−27.1	34.4	34.2	10.9	10.2
		Planar	4.6 (13.4)	−1.8 (14.2)	−22	−29.6	30.8	26	9.6	8.2
		ADL	8.4 (15)	8.8 (17.2)	−20.8	−24.6	38	42.4	12.2	12.2
	Elbow Flexion Extension	All Tasks	2.4	9.3	−34.3	−31.3	39.6	49.7	17.7	16.7
		Planar	1.4 (16.8)	9.8 (18)	−31.8	−25.6	35.2	44.8	15.8	13.8
		ADL	3.4 (20.8)	8.8 (23.2)	−36.8	−37	44	54.6	19.6	19.6
	Plane Of Elevation	All Tasks	15.9	46.7	−30.8	−7.5	63	100.9	19.4	19.3
		Planar	18.2 (23.8)	51.4 (26.8)	−28.4	−1.4	65.4	104	18.6	18
		ADL	13.6 (23.8)	42 (28.6)	−33.2	−13.6	60.6	97.8	20.2	20.6
	Rotation	All Tasks	−23.3	−50.9	−79	−106	32.4	3.8	21.8	20.7
		Planar	−30.4 (29.6)	−63.8 (27.6)	−88.4	−118.2	27.8	−9.4	20.2	18.4
		ADL	−16.2 (27.4)	−38 (28.4)	−69.6	−93.8	37	17	23.4	23
Sagittal	Angle Of Elevation	All Tasks	4.7	6.5	−18.4	−17.5	27.9	30.2	9.3	8.5
		Planar	3 (12)	3 (12.2)	−20.2	−21.6	26.4	27.2	8.8	7.6
		ADL	6.4 (11.6)	10 (11.6)	−16.6	−13.4	29.4	33.2	9.8	9.4
	Elbow Flexion Extension	All Tasks	6.7	13.3	−28.2	−21.7	41.3	48.8	16	13.8
		Planar	4.2 (18.6)	10 (19.4)	−32.2	−27.6	40.2	48.2	16.6	15
		ADL	9.2 (17)	16.6 (16.8)	−24.2	−15.8	42.4	49.4	15.4	12.6
	Plane Of Elevation	All Tasks	−14.9	23.6	−55.7	−27.1	25.9	74.6	15.8	15.9
		Planar	−13.2 (20.8)	26 (27.4)	−54	−27.6	27.2	79.6	14.4	15.6
		ADL	−16.6 (20.8)	21.2 (24.2)	−57.4	−26.6	24.6	69.6	17.2	16.2
	Rotation	All Tasks	5.1	−22.2	−50.8	−85.1	61.1	40.8	21.3	21.2
		Planar	0.8 (30.2)	−31.2 (32.4)	−57.8	−94.6	59.6	32.2	20.8	22.4
		ADL	9.4 (27.2)	−13.2 (32)	−43.8	−75.6	62.6	49.4	21.8	20

### 3.1. Joint angle agreement between Azure Kinect and OptiTrack

The Azure Kinect demonstrates moderate agreement with marker-based motion capture systems in measuring joint angles during upper limb activities of daily living, though this agreement varies considerably depending on the specific joint angle being measured and the complexity of the movement. The agreement between systems follows a clear hierarchy based on movement complexity. Angle of elevation shows the best agreement, with RMSE values as low as $8^{\circ }$ for OpenSim IK during planar movements. Elbow flexion-extension agreement had RMSE values of $13^{\circ }$ during ADLs. Plane of elevation shows moderate agreement with the RMSE values of $16^{\circ }$, while shoulder rotation measurements consistently show the highest disagreement with RMSE values of $21^{\circ }$.

### 3.2. Inverse kinematics effect on joint angle agreement

Inverse kinematics significantly improves the joint angle accuracy compared to the coordinate frame method when the Kinect is positioned with a view of the sagittal plane (f=6.79, p=0.009, Cohen’s f=0.91). Elbow flexion-extension exhibited the greatest improvement, with RMSE decreasing from $16.0^{\circ }$ using the coordinate frame method to $14^{\circ }$ with inverse kinematics. However, in the frontal plane, there was no significant difference between methods (f=1.17, p=0.279, Cohen’s f=0.04).

### 3.3. Camera positioning effect on joint angle agreement

The sagittal plane position provides significantly better agreement than the frontal plane position, representing the most important finding for practical implementation of Azure Kinect systems. Both measurement methods show statistically significant differences between viewing planes, with the Kinect coordinate frame method showing (f=8.97, p=0.003, Cohen’s f=0.10), and the OpenSim IK method showing(f=22.28, p<0.001, Cohen’s f=0.17).

## 4. Discussion

This study’s findings address three research questions regarding single-camera markerless motion capture (SCMoCap): the Azure Kinect’s agreement with OptiTrack, the benefits of OpenSim’s inverse kinematics (IK) over the coordinate frame method (CF), and the optimal camera placement. The Azure Kinect, when used with OpenSim IK, shows reasonable agreement with OptiTrack for simpler 2D motion patterns, particularly in angles like shoulder angle of elevation in the sagittal plane, with an average RMSE of approximately 8 ${\;}^{\circ }$, suggesting reliable capture of basic movements without extensive rotation or complex joint mechanics. However, Kinect struggles with 3D kinematics, such as tracking shoulder plane of elevation, especially in tasks with larger ranges of motion, and rotational measurements showed the largest discrepancies. The pattern of higher RMSE values for complex, multi-planar movements, such as shoulder rotationand activities like brushing hair or shoulder abduction, reflects the fundamental challenge that single-camera markerless motion capture faces with complex, multi-planar movements where depth perception becomes critical and joint center estimation becomes more difficult. This is likely due to the combination of large range of motion, multiple joint coordination, and potential occlusion issues inherent in single-camera systems.

Introducing model-based inverse kinematics through OpenSim improves joint angle agreement, with IK consistently outperforming CF across most joint angles. The significant improvement in elbow flexion-extension likely reflects how the global optimization of inverse kinematics distributes marker error across the kinematic chain (shoulder, elbow, wrist), in contrast to the coordinate frame method, which depends on accurate detection of each joint center in isolation. However, in the frontal plane, the lack of significant difference between methods highlights that biomechanical modeling and inverse kinematics cannot fully compensate for the limitations of single-camera depth sensing and joint localization, especially when camera placement is suboptimal or movements are out of plane.

Sagittal views outperformed frontal views across most joint angles, indicating that sagittal Kinect placement enhances accuracy. The superior results from sagittal positioning stem from several biomechanical and technical factors. Most upper limb activities of daily living primarily occur in the sagittal plane, as humans naturally reach forward, lift objects to their mouth, and perform functional tasks in this plane of motion. The side-viewing camera position captures these movements in their primary plane of motion, reducing the complexity of three-dimensional reconstruction. Additionally, the sagittal view minimizes self-occlusion issues that commonly occur when viewing from the front, where the torso can obstruct arm movements and where determining arm position relative to the body becomes more challenging. The sagittal position also provides better visualization of shoulder extension and flexion patterns that are fundamental to most functional activities, allowing the camera to more accurately track the natural movement patterns that characterize daily living tasks.

Across angle types, the Kinect coordinate frame method exhibited lower bias than the IK approach, indicating that systematic error is less prominent than random error in raw Kinect joint estimations. This suggests that Kinect’s measurements are more affected by noise – unpredictable fluctuations due to factors like sensor limitations or inaccuracies in joint detection – rather than a consistent offset. The high standard deviation relative to the bias reflects substantial variability in the measurements, even though the average systematic difference (bias) is low. This variability is evident in the large Limits of Agreement (LoA), meaning individual measurements can vary widely around the true value. Thus, evaluating the agreement of the entire signal, rather than just focusing on the range of motion, is essential to capture significant fluctuations that might otherwise be overlooked. In contrast, with OpenSim, the bias and RMSE are more closely aligned, showing a consistent offset from the OptiTrack measurements but with more predictable and stable errors. This alignment suggests that OpenSim is less influenced by random errors, making it more reliable in terms of accuracy, despite having a higher bias. OpenSim’s consistency in error suggests it is less prone to variability compared to the Kinect coordinate frame method. While this study focused on the accuracy of OpenSim’s IK tool rather than its usability, OpenSim provides a graphical user interface that facilitates biomechanical modelling; however, its complexity may pose challenges for non-technical clinical users without specialised training, potentially limiting its immediate accessibility in routine clinical practice. Additionally, as this study did not target-specific clinical conditions, a quantitative threshold such as a minimum clinically important difference (MCID) was not defined, which is a limitation in interpreting the clinical relevance of the results. Readers should interpret the findings based on their specific use case, considering the context of their clinical or research applications.

Özsoy et al. (2022) evaluated the agreement between a BTSSmart D×100Maker-based system and Azure Kinect, reporting a Bland-Altman analysis for shoulder flexion, abduction, internal rotation, and external rotation. Different Euler angle sequences were employed for the analysis: XZY rotation sequences were used for abduction, ZXY for flexion, and XZY sequences for rotations. The averages of the maximum and minimum values across five sequences were then calculated (cycle sequence mean), and the ROM was derived by calculating the difference between these mean maximum and minimum values. They reported biases of −4.0${\;}^{\circ }$, −9.6${\;}^{\circ }$, 4.6${\;}^{\circ }$, and −7.3${\;}^{\circ }$ for shoulder flexion, abduction, internal rotation, and external rotation, respectively, with LoA from −20.2${\;}^{\circ }$ to 12.3${\;}^{\circ }$, −31.9${\;}^{\circ }$ to 12.7${\;}^{\circ }$, −19.1${\;}^{\circ }$ to 28.2${\;}^{\circ }$, and −31.0${\;}^{\circ }$ to 16.4${\;}^{\circ }$. However, the movements were not recorded synchronously due to reported multipath interference issues, and only ROM agreement was reported.

The Azure Kinect shows significant improvement over the Azure Kinect’s predecessor. Galna et al. (Galna et al. 2014) measured the agreement between the Kinect V1 and a Vicon system with controls and Parkinson’s patients during a series of clinically relevant Parkinson’s disease (PD) movements. Based on the range of motion, they reported biases of 16.93${\;}^{\circ }$, 8.68${\;}^{\circ }$, and 10.44${\;}^{\circ }$ for elbow flexion, shoulder abduction, and shoulder flexion, respectively, with LoA of 17.98${\;}^{\circ }$, 9.82${\;}^{\circ }$, and 5.15${\;}^{\circ }$, in control subjects. For Parkinson’s disease patients, they reported biases of 21.66${\;}^{\circ }$, 11.57${\;}^{\circ }$, and 15.02${\;}^{\circ }$ (elbow flexion, shoulder abduction, and shoulder flexion, respectively), with LoA of 44.58${\;}^{\circ }$, 22.86${\;}^{\circ }$, and 11.57${\;}^{\circ }$. It is worth noting that they found the agreement to be worse for PD patients. There is no evidence to suggest that this difference is mitigated with the Azure Kinect, and this issue is also likely to apply to most other popular pose estimation algorithms, such as OpenPose (Cao et al. 2019), that were trained on publicly available datasets such as COCO (Lin et al. 2014) or MPII (Andriluka et al. 2014), due to a lack of pathology-specific training data. Solutions that utilise biomechanical modelling, such as OpenCap (Uhlrich et al. 2023), may offer more generalised pose estimation for pathological movement as they report state-of-the-art agreement when measuring healthy populations simulating pathological movement; however, this has not yet been verified with any patient populations and it is also a multi-camera system.

Difficulty in estimating forearm pronation-supination was found across studies. Galna et al. (2014) reported that the Kinect V1 did not allow for the measurement directly and instead calculated the vertical displacement of the wrist as a proxy. Scano et al. (2020) found that poor thumb tracking was responsible for the inaccurate estimation of this movement. These results align with our findings and suggest that the Azure Kinect still lacks the accuracy necessary to track the thumb at a distance and thus cannot measure pronation-supination.

The Intel real-sense d435 (Intel, USA) is often compared with the azure Kinect due to their similar features and relatively low cost. The d435 offers stereo depth, rather than IR depth, which means it can be used in synchronised data collection without interference issues. Van Crombrugge et al. (2022) evaluated the agreement of joint angles between the d435 markerless and a Vicon maker-based system using the whole signal. They reported RMSEs for elbow flexion (32°), shoulder abduction (28°), and shoulder flexion (41°). Our results show that the Azure Kinect has better agreement than this. One reason for this may be the d435’s lower quality depth map (Clarke and Mills 2021).

Needham et al. (2021) compared a 9-camera OpenPose markerless setup with an Oqus (Qualysis AB, Gothenburg, Sweden) marker-based system during walking, running and jumping. They reported that shoulder joint center locations fell within the known range of error for marker-based motion capture. While OpenPose is considered one of the most accurate and robust open-source solutions for markerless motion capture, there is limited evidence assessing its joint angle agreement with marker-based systems for upper limb tasks. Most studies focus on joint center locations during gait, which is difficult for clinicians to interpret, as they are primarily interested in understanding error in terms of joint angles (Schwartz et al. 2004).

Some trials were excluded due to the Kinect body tracking algorithm mistaking a jacket on a chair for a person during one trial. This exclusion highlights the limitations of the Kinect algorithm, which relies on a convolutional neural network (CNN) to process each frame independently. The CNN is responsible for detecting and estimating body joints, by analysing the RGB and depth data from each frame. After processing each frame, the system uses additional post-processing steps to link these detected joints across frames, thereby maintaining continuity in tracking individuals over time. However, the CNN can struggle in certain scenarios, particularly when the visual input is ambiguous or contains unexpected objects, such as mistaking a jacket on a chair for a person. This initial misinterpretation by the CNN can lead to further difficulties in maintaining accurate tracking across frames during post-processing. Such challenges have been noted by other users in the Kinect SDK GitHub and documented in the literature, indicating that the system’s ability to correctly identify and track subjects can be compromised in less structured environments. Despite the Azure Kinect’s discontinuation, this study establishes a comprehensive reference framework for evaluating depth-based single-camera markerless motion capture systems. By providing a novel methodology that integrates synchronous data collection, inverse kinematics via OpenSim, and clinically interpretable joint angle measurements for upper limb tasks, it offers a robust standard for assessing improvements in successors like the Femto Bolt (ORBBEC, USA). This framework enables researchers and clinicians to compare the performance of emerging SCMoCap systems against our findings, facilitating advancements in accessible, cost-effective motion analysis for applications such as at-home rehabilitation and clinical decision-making for patients with mobility impairments.

## 5. Conclusion

This study demonstrates that the Azure Kinect, a single-camera markerless motion capture (SCMoCap) system, when integrated with OpenSim’s inverse kinematics (IK), achieves reasonable accuracy in measuring some upper limb joint angles compared to the gold standard OptiTrack system, particularly for simpler motions like shoulder elevation (8 ${\;}^{\circ }$) and elbow flexion-extension (14 ${\;}^{\circ }$) in the sagittal plane. The IK method outperformed the coordinate frame approach; however, limitations in tracking complex 3D motions, such as shoulder rotation and forearm pronation-supination, highlight the need for enhanced landmark detection to fully utilize model-based inverse kinematics.

The findings highlight SCMoCap’s potential to broaden access to motion analysis, reducing reliance on specialized, resource-intensive marker-based systems or the complex setup of multi-camera markerless systems, and enabling applications in diverse settings, such as at-home rehabilitation. By leveraging biomechanical modelling, this approach enhances the clinical interpretability of joint angle data, supporting better informed treatment decisions. Future research should prioritise improving pose estimation for upper limb tasks, validating SCMoCap with clinical populations, and evaluating emerging devices like the Femto Bolt. These advancements could transform motion capture into a widely accessible tool, empowering clinicians to deliver more targeted and data driven interventions, ultimately improving outcomes for patients with mobility impairments.

## Supplementary Material

Supplemental Material

## References

[cit0001] Andriluka M, Pishchulin L, Gehler P, Schiele B. 2014. 2D human pose estimation: new benchmark and state of the art analysis. 2014 IEEE Conference on Computer Vision and Pattern Recognition; Columbus, OH, USA. [accessed 2024 10 7]. 3686–3693. https://ieeexplore.ieee.org/document/6909866/?arnumber=6909866.

[cit0002] Arnold AS, Liu MQ, Schwartz MH, Õunpuu S, Delp SL. 2006. The role of estimating muscle-tendon lengths and velocities of the hamstrings in the evaluation and treatment of crouch gait. Gait Posture. 23(3):273–281. [accessed 2024 10 31]. https://www.sciencedirect.com/science/article/pii/S0966636205000627. 15964759 10.1016/j.gaitpost.2005.03.003

[cit0003] Ben Gamra M, Akhloufi MA. 2021. A review of deep learning techniques for 2D and 3D human pose estimation. Image Vision Comput. 114:104282. [accessed 2024 04 27]. https://www.sciencedirect.com/science/article/pii/S0262885621001876.

[cit0004] Cao Z, Hidalgo G, Simon T, Wei SE, Sheikh Y. 2019. OpenPose: realtime multi-person 2D pose estimation using part affinity fields; May. ArXiv:1812.08008 [cs]; [accessed 2024 04 27]. http://arxiv.org/abs/1812.08008. 10.1109/TPAMI.2019.2929257 31331883

[cit0005] Capecci M, Ceravolo MG, Ferracuti F, Grugnetti M, Iarlori S, Longhi S, Romeo L, Verdini F. 2018. An instrumental approach for monitoring physical exercises in a visual markerless scenario: a proof of concept. J Biomech. 69:70–80. doi: 10.1016/j.jbiomech.2018.01.008. 29398000

[cit0006] Chadwick EK, Blana D, Kirsch RF, van den Bogert AJ. 2014. Real-time simulation of three-dimensional shoulder girdle and arm dynamics. IEEE Trans Biomed Eng. 61(7):1947–1956. Conference Name: IEEE Transactions on Biomedical Engineering; [accessed 2023 10 27]. https://ieeexplore.ieee.org/document/6755458. 24956613 10.1109/TBME.2014.2309727 PMC4068297

[cit0007] Clarke J, Mills S. 2021 Dec. Sensor evaluation for voxel-based RGB-D SLAM. 2021 36th International Conference on Image and Vision Computing New Zealand (IVCNZ). Tauranga, New Zealand: IEEE. p. 1–6. [accessed 2023 10 27]. https://ieeexplore.ieee.org/document/9653223/.

[cit0008] Corazza S, Mündermann L, Chaudhari AM, Demattio T, Cobelli C, Andriacchi TP. 2006. A markerless motion capture system to study musculoskeletal biomechanics: visual hull and simulated annealing approach. Ann Biomed Eng. 34(6):1019–1029. [accessed 2022 01 13]. 10.1007/s10439-006-9122-8. 16783657

[cit0009] Delp SL, Anderson FC, Arnold AS, Loan P, Habib A, John CT, Guendelman E, Thelen DG. 2007. Opensim: open-source software to create and analyze dynamic simulations of movement. IEEE Trans Biomed Eng. 54(11):1940–1950. [accessed 2023 10 27]. https://ieeexplore.ieee.org/abstract/document/4352056?casa_token=BRcH4SeitJoAAAAA:EVBLtypfJZ7UkaWMmhP-phBxnpGc5Ebke9VAmvSeI7pb33mjZgZjdwlgUaLdaHgmhUVuBf4eQ2r81g. 18018689 10.1109/TBME.2007.901024

[cit0010] Ehrig RM, Taylor WR, Duda GN, Heller MO. 2006. A survey of formal methods for determining the centre of rotation of ball joints. J Biomech. 39(15):2798–2809. doi: 10.1016/j.jbiomech.2005.10.002. 16293257

[cit0011] Eltoukhy M, Kuenze C, Oh J, Jacopetti M, Wooten S, Signorile J. 2017. Microsoft Kinect can distinguish differences in over-ground gait between older persons with and without Parkinson’s disease. Med Eng Phys. 44:1–7. [accessed 2021 06 17]. https://linkinghub.elsevier.com/retrieve/pii/S1350453317300887. 28408157 10.1016/j.medengphy.2017.03.007

[cit0012] Galna B, Barry G, Jackson D, Mhiripiri D, Olivier P, Rochester L. 2014. Accuracy of the Microsoft Kinect sensor for measuring movement in people with Parkinson’s disease. Gait Posture. 39(4):1062–1068. [accessed 2022 10 1]. https://www.sciencedirect.com/science/article/pii/S0966636214000241. 24560691 10.1016/j.gaitpost.2014.01.008

[cit0013] Kanko RM, Laende EK, Davis EM, Selbie WS, Deluzio KJ. 2021. Concurrent assessment of gait kinematics using marker-based and markerless motion capture. J Biomech. 127:110665. [accessed 2021 10 31]. https://www.sciencedirect.com/science/article/pii/S0021929021004346. 34380101 10.1016/j.jbiomech.2021.110665

[cit0014] Kidziński Yang B, Hicks JL, Rajagopal A, Delp SL, Schwartz MH. 2020. Deep neural networks enable quantitative movement analysis using single-camera videos. Nat Commun. 11(1):4054. [accessed 2025 01 8]. https://www.nature.com/articles/s41467-020-17807-z. 32792511 10.1038/s41467-020-17807-z PMC7426855

[cit0015] Laracca E, Stewart C, Postans N, Roberts A. 2014. The effects of surgical lengthening of hamstring muscles in children with cerebral palsy – the consequences of pre-operative muscle length measurement. Gait Posture. 39(3):847–851. [accessed 2024 10 31]. https://www.sciencedirect.com/science/article/pii/S0966636213006826. 24332744 10.1016/j.gaitpost.2013.11.010

[cit0016] Lin TY, Maire M, Belongie S, Hays J, Perona P, Ramanan D, Dollár P, Zitnick CL. 2014. Microsoft COCO: common objects in context. In: Fleet D, Pajdla T, Schiele B Tuytelaars T, editors. Computer vision – ECCV 2014. Zurich, Switzerland: Springer International Publishing; p. 740–755. Lecture Notes in Computer Science.

[cit0017] Naeemabadi M, Dinesen B, Andersen OK, Hansen J. 2018. Investigating the impact of a motion capture system on Microsoft Kinect v2 recordings: a caution for using the technologies together. PLOS ONE. 13(9):e0204052. [accessed 2022 08 11]. https://journals.plos.org/plosone/article?id=10.1371/journal.pone.0204052. 30216382 10.1371/journal.pone.0204052 PMC6157830

[cit0018] Needham L, Evans M, Cosker DP, Wade L, McGuigan PM, Bilzon JL, Colyer SL. 2021. The accuracy of several pose estimation methods for 3D joint centre localisation. Sci Rep. 11(1):20673. [accessed 2024 08 12]. https://www.nature.com/articles/s41598-021-00212-x. 34667207 10.1038/s41598-021-00212-x PMC8526586

[cit0019] Noort J, Kortier HG, Beek N, Veeger DHEJ, Veltink PH. 2016. Measuring 3D hand and finger kinematics—a comparison between inertial sensing and an opto-electronic marker system. PLOS ONE. 11(11):e0164889. [accessed 2022 01 13]. https://journals.plos.org/plosone/article?id=10.1371/journal.pone.0164889. 27812139 10.1371/journal.pone.0164889 PMC5094774

[cit0020] Osborne M, Mueske NM, Rethlefsen SA, Kay RM, Wren TAL. 2019. Pre-operative hamstring length and velocity do not explain the reduced effectiveness of repeat hamstring lengthening in children with cerebral palsy and crouch gait. Gait Posture. 68:323–328. [accessed 2024 04 23]. https://www.sciencedirect.com/science/article/pii/S0966636218307434. 30572181 10.1016/j.gaitpost.2018.11.033 PMC6370486

[cit0021] Özsoy U, Yıldırım Y, Karaşin S, Şekerci R, Süzen LB. 2022. Reliability and agreement of Azure Kinect and Kinect v2 depth sensors in the shoulder joint range of motion estimation. J Shoulder And Elb Surg. 31(10):2049–2056. [accessed 2024 09 7]. https://www.jshoulderelbow.org/article/S1058-2746(22)00434-7/abstract. 10.1016/j.jse.2022.04.007 35562032

[cit0022] Philp F, Faux-Nightingale A, Woolley S, de Quincey E, Pandyan A. 2021. Implications for the design of a diagnostic decision support system (DDSS) to reduce time and cost to diagnosis in paediatric shoulder instability. BMC Med Inf Decis Mak. 21(1):78. [accessed 2024 10 21]. 10.1186/s12911-021-01446-5. PMC7912970 33639920

[cit0023] Philp F, Freeman R, Stewart C. 2022. An international survey mapping practice and barriers for upper-limb assessments in movement analysis. Gait Posture. 96:93–101. [accessed 2024 12 20]. https://www.sciencedirect.com/science/article/pii/S0966636222001515. 35623317 10.1016/j.gaitpost.2022.05.018

[cit0024] Sá F, Marques A, Rocha NBF, Trigueiro MJ, Campos C, Schröder J. 2015. Kinematic parameters of throwing performance in patients with schizophrenia using a markerless motion capture system. Somatosens Mot Res. 32(2):77–86. doi: 10.3109/08990220.2014.969838. 25365543

[cit0025] Salami F, Brosa J, Van Drongelen S, Klotz MCM, Dreher T, Wolf SI, Thielen M. 2019. Long-term muscle changes after hamstring lengthening in children with bilateral cerebral palsy. Develop Med Child Neuro. 61(7):791–797. [accessed 2024 04 23]. https://onlinelibrary.wiley.com/doi/abs/10.1111/dmcn.14097. 10.1111/dmcn.14097 30474110

[cit0026] Scano A, Mira RM, Cerveri P, Molinari Tosatti L, Sacco M. 2020. Analysis of upper-limb and trunk kinematic variability: accuracy and reliability of an RGB-D sensor. Multimodal Technol Interact. 4(2):14. [accessed 2024 10 24]. https://www.mdpi.com/2414-4088/4/2/14.

[cit0027] Schwartz MH, Trost JP, Wervey RA. 2004. Measurement and management of errors in quantitative gait data. Gait Posture. 20(2):196–203. [accessed 2025 01 8]. https://www.sciencedirect.com/science/article/pii/S0966636203001681. 15336291 10.1016/j.gaitpost.2003.09.011

[cit0028] Scott B, Seyres M, Philp F, Chadwick EK, Blana D. 2022. Healthcare applications of single camera markerless motion capture: a scoping review. PeerJ. 10:e13517. doi: 10.7717/peerj.13517. 35642200 PMC9148557

[cit0029] Seo NJ, Fathi MF, Hur P, Crocher V. 2016. Modifying kinect placement to improve upper limb joint angle measurement accuracy. J Hand Ther. 29(4):465–473. doi: 10.1016/j.jht.2016.06.010. 27769844 PMC6701865

[cit0030] Stone EE, Skubic M. 2015. Fall detection in homes of older adults using the Microsoft Kinect. IEEE J Biomed Health inform. 19(1):290–301. [accessed 2025 01 8]. https://ieeexplore.ieee.org/document/6774430. 24733032 10.1109/JBHI.2014.2312180

[cit0031] Topka H, Konczak J, Dichgans J. 1998. Coordination of multi-joint arm movements in cerebellar ataxia:. Exp Brain Res. 119(4):483–492. [accessed 2022 01 13]. 10.1007/s002210050364. 9588783

[cit0032] Uhlrich SD, Falisse A, Kidziński Muccini J, Ko M, Chaudhari AS, Hicks JL, Delp SL. 2023. Opencap: human movement dynamics from smartphone videos. PLoS Comput Biol. 19(10):e1011462. [accessed 2024 04 23]. https://journals.plos.org/ploscompbiol/article?id=10.1371/journal.pcbi.1011462. 37856442 10.1371/journal.pcbi.1011462 PMC10586693

[cit0033] Van Crombrugge I, Sels S, Ribbens B, Steenackers G, Penne R, Vanlanduit S. 2022. Accuracy assessment of joint angles estimated from 2D and 3D camera measurements. Sensors. 22(5):1729. [accessed 2022 06 23]. https://www.mdpi.com/1424-8220/22/5/1729. 35270875 10.3390/s22051729 PMC8914870

[cit0034] Wade DT, Collin C. 1988. The Barthel ADL index: a standard measure of physical disability? Int Disabil Stud. 10(2):64–67. doi: 10.3109/09638288809164105. 3042746

[cit0035] Wade L, Needham L, Evans M, McGuigan P, Colyer S, Cosker D, Bilzon J, Gu Y. 2023. Examination of 2d frontal and sagittal markerless motion capture: implications for markerless applications. PLOS ONE. 18(11):e0293917. [accessed 2024 07 30]. https://journals.plos.org/plosone/article?id=10.1371/journal.pone.0293917. 37943887 10.1371/journal.pone.0293917 PMC10635560

[cit0036] Wu G, van der Helm FCT, Veeger HEJD, Makhsous M, Van Roy P, Anglin C, Nagels J, Karduna AR, McQuade K, Wang X, et al. 2005. ISB recommendation on definitions of joint coordinate systems of various joints for the reporting of human joint motion–part II: shoulder, elbow, wrist and hand. J Biomech. 38(5):981–992. doi: 10.1016/j.jbiomech.2004.05.042. 15844264

[cit0037] Zheng C, Wu W, Chen C, Yang T, Zhu S, Shen J, Kehtarnavaz N, Shah M. 2023. Deep learning-based human pose estimation: a survey. ACM Comput Surv. 56(1):1–37. [accessed 2024 12 20]. https://dl.acm.org/doi/10.1145/3603618.

