# Supplementary figures and images for: Assessing single camera markerless motion capture with OpenSim inverse kinematics during upper limb activities of daily living
Source: Int Biomech. 2025 Sep 5;12(1):1–13. doi: 10.1080/23335432.2025.2556187 (PMC12416023; doi:10.1080/23335432.2025.2556187)

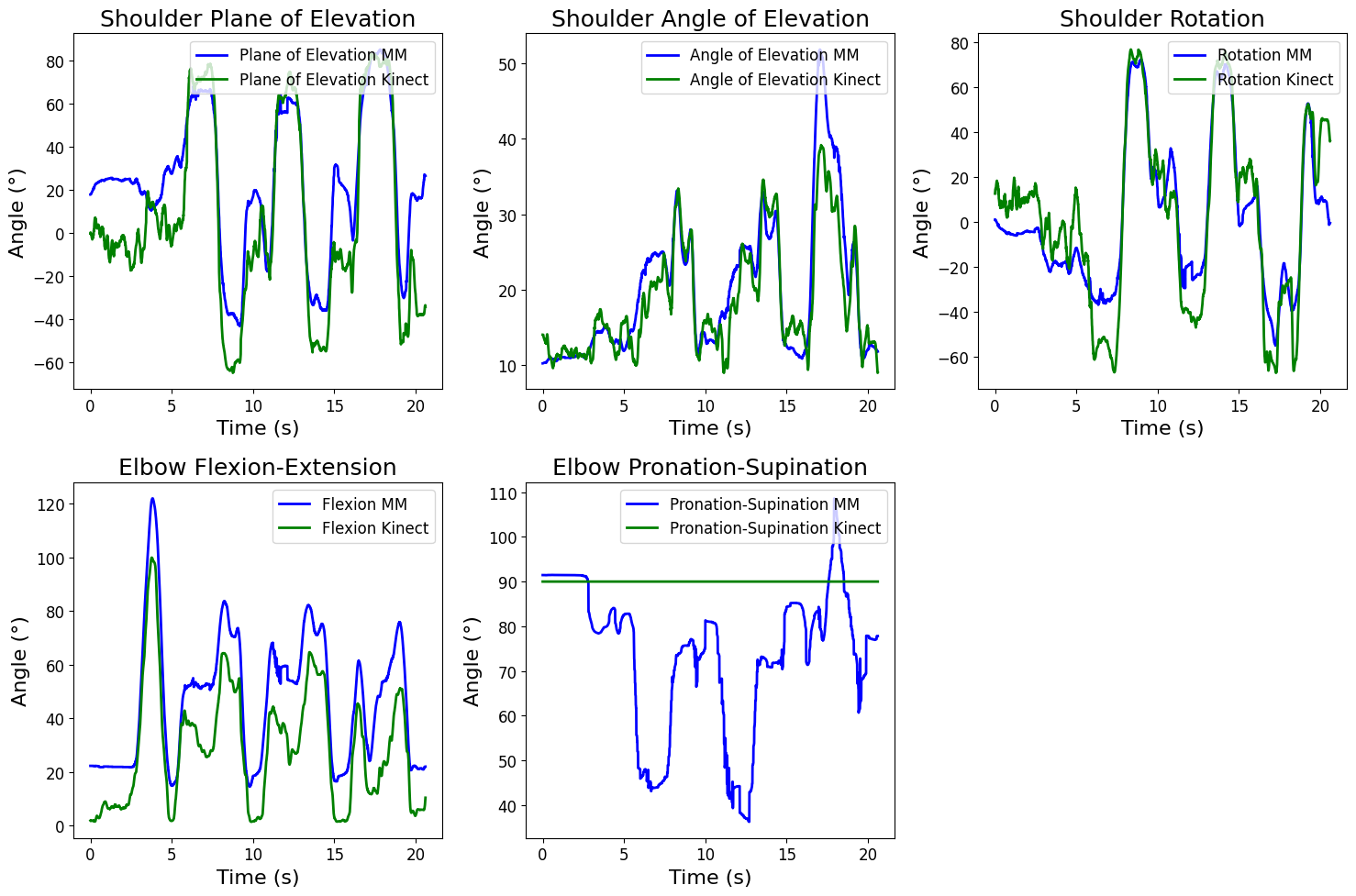

Supplement: Supplemental Material [file TBBE_A_2556187_SM3716.zip › Supplementary Material/supplementary_figure_1_example_collect_change_sagittal_ik.png]
